# Preparation Optimization of Enhanced Poplar Wood by Organic–Inorganic Hybrid Treatment via Response Surface Methodology

**DOI:** 10.3390/ma16206718

**Published:** 2023-10-17

**Authors:** Yong Wang, Xia He, Layun Deng, Xiazhen Li, Xianjun Li

**Affiliations:** 1 College of Material Science and Engineering, Central South University of Forestry and Technology, Changsha 410004, China; satanwy@163.com (Y.W.); lixiazhen198500@163.com (X.L.); 2 State Key Laboratory of Utilization of Woody Oil Resource, Hunan Academy of Forestry, Changsha 410004, China; deng269@163.com; 3 College of Mechanical and Electrical Engineering, Hunan City University, Yiyang 413000, China; hexia1990@foxmail.com

**Keywords:** poplar wood, hybrid treatment, surface-modified montmorillonite, response surface methodology

## Abstract

In this work, a strategy for hybrid treatment was proposed, aiming to present a hybrid impregnation agent including lignin-derived resin (LR) and surface-modified montmorillonite (GMMT) to treat fast-growing poplar wood. The treating agents could penetrate the wood, fill the cavities of the wood interior, and strengthen the cell wall structure. The optimal WPG of 36.2% was obtained upon the response surface methodology (RSM) at the conditions of 34% LR, 1.8% GMMT, 1.2 MPa impregnation pressure, and 99 min impregnation time. The density, water uptake (WU), modulus of rupture (MOR), modulus of elasticity (MOE), and compressive strength (CS) of the samples were tested to evaluate the enhancement of the physical and mechanical properties. In addition, these samples were investigated via cone calorimeter (CONE), Fourier Transform Infrared spectrometer (FTIR), and X-ray diffraction (XRD). The results showed that the density of the treated samples increased significantly up to 0.72 g/cm^3^. Compared with 134.8% of the control, the WU of the treated wood sample could decrease to 60.3%. In addition, the MOR and MOE of the resulting samples reached up to 131.8 MPa and 18.14 GPa, respectively, which were 62.3% and 77.7% higher than the control. Notably, the CS was 84.7 MPa with an increase of up to 94.7%. Moreover, the peak heat release rate (HRR) of the treated sample was obviously reduced to 231.33 kW/m^2^, a decrease of 17.5% compared to the control (271.71 kW/m^2^).

## 1. Introduction

Fast-growing wood as a natural resource could be used as building materials with the advantages of short growing cycles and high storage capacity [1,2]. It can be an alternative to natural forest wood and is suitable for large-scale logging and applications. However, due to its poor mechanical and physical properties, fast-growing wood could not be used widely, and its service life is short. Various methods have been applied to enhance the performance of fast-growing wood [3,4]. Chemical treatment has proven to be effective in the improvement in fast-growing wood with increased mechanical and physical properties by reducing the hydrophilic groups, filling the wood cavities, and strengthening the cell wall structures [5,6]. However, most of the treating chemicals involved are toxic, which may cause environmental pollution and affect human health [7,8]. Resin impregnation of wood as one of the chemical treatment methods has the advantage of simplified procedure and low toxic resins. The procedures that have been generally used so far are mainly urea-formaldehyde resin [9], phenolic resin [10], and some synthetic resins [11,12,13]. However, despite excellent performance in wood treatment, most of these resins are derived from petroleum-based chemicals, which are non-renewable. Therefore, bio-based alternatives from natural and renewable resources are important for wood treatment. Bio-based resins, such as furfuryl alcohol (FA), have been widely studied and used in recent years [14,15]. However, the conversion process of FA is complicated and expensive. Cost-effective bio-based resins are desirable and imperative for wood treatment.

Lignin is a component of biomass materials, which is abundant, easily available, and especially environmentally friendly. Most of the lignin material is used as fuel or discharged as black liquid waste, which hinders the carbon cycle in the Earth’s biosphere and causes a loss of potential multi-functional raw materials. In recent years, it has gained increasing attention for its applications in the preparation of bio-based chemicals due to its non-toxic and renewable properties. The units of lignin are aromatic monomers, including sinapyl alcohols, coniferyl alcohols, and p-coumaryl alcohols [16]. These units can be used as an alternative to partly or even completely replace the phenol for the synthesis of bio-based PF. The lignin-based PF (LR) has been studied extensively for applications of wood panel adhesives, but there are few reports about the application of wood treatment. Similar to other resin impregnations, the LR impregnation is promising for the improvement in the mechanical and physical properties [3,17]. However, the thermal stability is poor and could be easily burned during combustion. In this context, to enhance thermal stability and retardancy, an organic–inorganic hybrid treatment was proposed to combine resin impregnation and inorganic particle filling [18].

Montmorillonite (MMT) clay is especially suitable for wood treatment, which is natural, cheap, and easily available for wide applications around the world. In addition, the studies of wood treatment involving MMT presented desirable effects, especially the improvement in thermal stability and fire retardancy. However, the compatibility of organic resins and inorganic particles is poor [19,20]. Simple blending of the two components could make it difficult to maintain desirable storage time due to the rapid precipitation of the inorganic particles in the polar solutions. Surface modification has been proven to be effective in converting the surface of organic particles into non-polar surfaces, which can then be compatible with the organic components [21,22]. The application of surface-modified GMMT to the wood treatment is promising. Therefore, combined with GMMT, LR could have significant effects on the comprehensive enhancement of fast-growing wood.

In this study, fast-growing poplar wood samples were treated with hybrid impregnation agents, including LR and GMMT, via the vacuum-impregnation process and heat-cured. The treating procedures were optimized using RSM experiments. Afterward, the as-prepared wood samples were characterized by Fourier Transform Infrared spectrometer (FTIR) and X-ray diffraction (XRD). Furthermore, the density, weight percent gain (WPG), water uptake (WU), modulus of elastic (MOE), modulus of rupture (MOR), and compressive strength (CS) of the resulting samples were tested to evaluate the effects of the treating process. Moreover, the combustion properties of the resulting samples were also investigated.

## 2. Materials and Methods

### 2.1. Materials

Poplar wood samples (Populus deltoides Marsh.) were purchased from Hunan Fusen Wood Industry (Yiyang, China). LR, EtOH, 3-Glycidoxy-propyltrimethoxysilane (KH570), and MMT used in this paper were provided by Xilong Chemical Reagent Co., Ltd., Shantou, China. The chemicals were all of analytical purity.

### 2.2. Preparations of GMMT

The method of surface modification of MMT was referred to in the study of Fu et al. [21]. To an EtOH solution, 5 wt% of MMT was dispersed (0.2 g/mL). The MMT/EtOH suspension was charged into a three-necked flask with 20 wt% of 3-Glycidoxy-propyltrimethoxysilane (KH570). The mixture was refluxed at 90 °C for 2 h with an N_2_ inlet. Finally, the reaction mixture was centrifuged and washed thoroughly with ethanol to remove the unreacted KH570, which was then put into a vacuum oven at 45 °C until it reached a constant weight. The obtained powder was the resultant GMMT.

### 2.3. Treatment of Poplar Wood Samples

The LR solution (25 wt%, 30 wt%, and 35 wt%) was prepared, and GMMT was added to the LR solution accompanying rapid stirring for 1 h to obtain the mixture, which was assigned as the LR/GMMT solution. For comparison, following exactly the same procedure, an LR/MMT solution was prepared. The as-prepared impregnation solutions were applied to the process of poplar wood modification.

The poplar wood samples were dried at 105 °C to a constant weight and were first put into a tank for vacuum at −0.1 MPa for 60 min. After the vacuum process, the impregnation solution was transported through a hose by negative pressure into the tank. When it reached the atmospheric pressure, the tank was pressurized to a fixed value for a while to press the impregnation solution into the wood for a fixed time (80 min, 90 min, and 100 min). After impregnation, the samples were put into an oven at 120 °C for 2 h.

The pristine poplar wood was assigned the name of the control. Based upon the optimum treating condition from RSM, unless otherwise specified, the samples treated with the LR (36 wt%) solution were denoted as LRW. Samples treated with 36 wt% LR and 1.8 wt% MMT were denoted as LRW/MMT, which were assigned as LRW/GMMT with 1.8 wt% GMMT.

### 2.4. RSM Experimental Design

The RSM was used to optimize the treating conditions of the poplar wood samples, including LR, GMMT, impregnation pressure, and impregnation time. The factor levels and coding are illustrated in Table 1.

Box–Behnken Design (BBD) was applied to investigate the interactions among the four factors with the weight percent gain (WPG) as the response Y. The following Equation (1) was used to calculate the value of WPG.

(1) $$\mathrm{WPG}=\frac{W_{t}-W_{0}}{W_{0}}\times 100\%$$

W_0_ is the initial dry weight of the wood sample and W_t_ is the final dry weight of the treated wood sample.

### 2.5. Characterizations of Samples

FTIR spectroscopic data were recorded in a NICOLET-is5 spectrometer (Thermo Fisher Scientific, Waltham, MA, USA) using an attenuated total reflectance (ATR) accessory. The flammability properties of the samples were measured by following the ISO 5660-1 standard [23] with an FTT0007 cone calorimeter (Fire Testing Technology Ltd., East Grinstead, UK). The upper face of test samples of 100 mm × 100 mm × 10 mm (R × T × L) were exposed under a heat flux of 75 kW/m^2^ in the horizontal position with the other sides wrapped in aluminum foil. An X-ray 6000 diffraction (Shimadzu, Osaka, Japan) machine was used to test the crystallization of the control and treated samples. The scanning rate was 1°/min, ranging from 0.5° to 40° at 30 rpm.

### 2.6. Physical and Mechanical Properties

The physical and mechanical performance (Density, WU, MOR, MOE, and CS) of the control, LRW, LRW/MMT, and LRW/GMMT were investigated. The sampling methods of poplar wood samples complied with the Chinese National Standard GB/T 1927.2-2021 [24]. The density of samples with the dimension of 2 cm × 2 cm× 2 cm (length × width × thickness) was tested in accordance with the Chinese National Standard GB/T 1927.5-2021 [25]. MOR and MOE of the samples with the dimension of 30 cm × 2 cm× 2 cm (length × width × thickness) were measured in accordance with the Chinese National Standard GBT1927.10-2021 [26]. The CS of all samples was investigated in accordance with the Chinese National Standard GB/T 1927.11-2021 [27] with the dimension of 3 cm × 2 cm× 2 cm (length × width × thickness). All these samples of mechanical tests were conserved under the condition of (20 ± 2) °C and (65 ± 3) %RH to reach 12% of equilibrium moisture content (EMC) before tests and carried out with six replicates.

The investigation of WU referred to the Chinese National Standard GB/T 1927.7-2021 [28]. The specific water temperature of the WU test was (20 ± 2) °C. The weights of the samples should be recorded when they were immersed in water for 6 h and 24 h, respectively. Then, the weights of the samples should be recorded at 1, 2, 4, 8, 12, and 20 days each. Afterwards, the samples should be weighed every 10 days. The WU of the test samples was subsequently calculated based on the initial dry weight and the wet weight recorded at a specific time. When the error in WU between two adjacent times was less than 5%, it could be considered that the samples were saturated by water.

## 3. Results and Discussion

### 3.1. Analysis of Variance (ANOVA) and Model Fitting

The experimental results of the BBD design are displayed in Table 2. The second-order polynomial equation presented below can calculate the coefficient of independent variables.

Y = 33.82 + 2.88A + 0.8B + 1.13C + 1.02D − 0.6AB − 1.13AC − 0.37AD + 0.35BC − 0.1BD − 0.05CD − 0.87A^2^ + 0.032B^2^ − 0.53C^2^ + 0.094D^2^ (2)

The results of the ANOVA analysis are presented in Table 3. The fitness quality of the predictive model is at an obviously significant level (*p* < 0.01). The lack of fit is 0.1527, demonstrating the credibility of the regression model to describe the relationship between the independent factors and the response. In addition, the determination coefficient R^2^ of the predictive model is 0.9679. The conclusion can be drawn from the ANOVA analysis that the regression model to describe the relationship between the independent factors and the response is credible.

**Figure 1:**
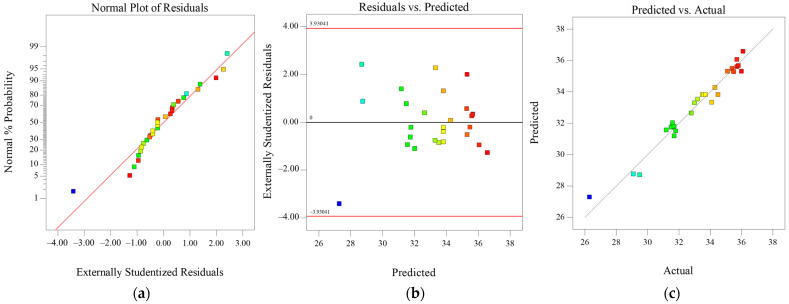
Normal plots of (**a**) residuals, (**b**) residuals vs. predicted, and (**c**) predicted vs. actual WPG of poplar wood samples.

Figure 1 shows the normal probability distribution of residuals, residuals vs. predicted, and predicted vs. actual values of WPG of poplar wood samples. The normal distribution diagram of WPG of the treating wood samples is basically on a straight line, indicating that the distribution of the residuals belongs to a normal distribution. At the same time, the distribution of residual error and predicted value has no obvious rule, and the predicted value and actual value of WPG of the treating wood samples are basically in a straight line, which also can be recognized as a normal distribution. Therefore, it can be further shown that the regression model and variance model are at a highly significant level (*p* < 0.05) with a good degree of fitting. The regression model is established and suitable for reasonable prediction of the effectiveness of impregnation modification processes.

**Figure 2:**
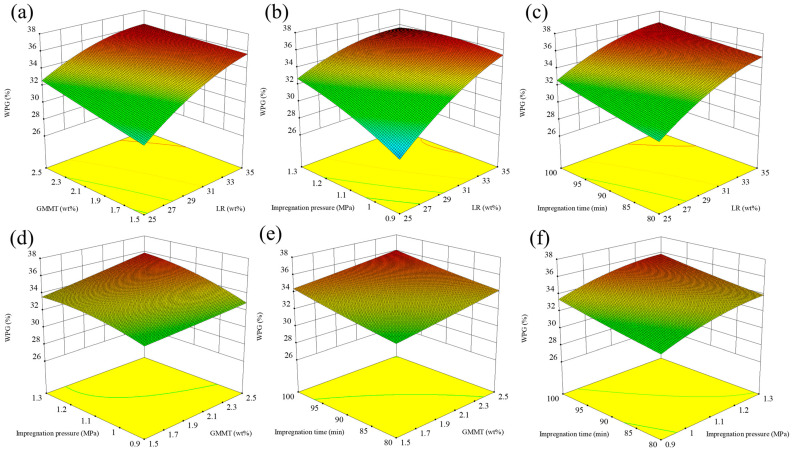
Contour plots for interaction effects of independent factors on WPG of treating poplar wood samples: (**a**) GMMT and LR; (**b**) Impregnation pressure and LR; (**c**) Impregnation time and LR; (**d**) Impregnation pressure and GMMT; (**e**) Impregnation time and GMMT; (**f**) Impregnation time and Impregnation pressure.

The 3D contour plots between the independent and dependent variables are illustrated in Figure 2. The independent factors have very significant effects (*p* < 0.01) on WPG in accordance with the 3D response surface curves. The interactions between the GMMT amount and the impregnation time and impregnation pressure were not significant. Notably, LR and impregnation pressure have a marked effect on WPG (*p* < 0.01). The 3D contour plots between the independent and dependent variables are consistent with the ANOVA analysis. Via the analysis and calculation of the fitting equation, the predicted WPG of the treated poplar wood was 36.2%. The corresponding optimal treating conditions were 34.12% LR, 1.76% GMMT, 1.15 MPa impregnation pressure, and 98.9 min impregnation time. The conditions of LR, GMMT, impregnation pressure, and impregnation time were rounded to 34%, 1.8%, 1.2 MPa, and 99 min, respectively. Based on the optimal conditions, three tests were performed to determine the WPG of the treated poplar wood. After the validation test, the average WPG of the validation tests was 34.9%, and the error rate between the predicted and measured values was 3.59% within limits, which verified the reliability of the response surface optimization model.

### 3.2. Physical and Mechanical Properties

**Figure 3:**
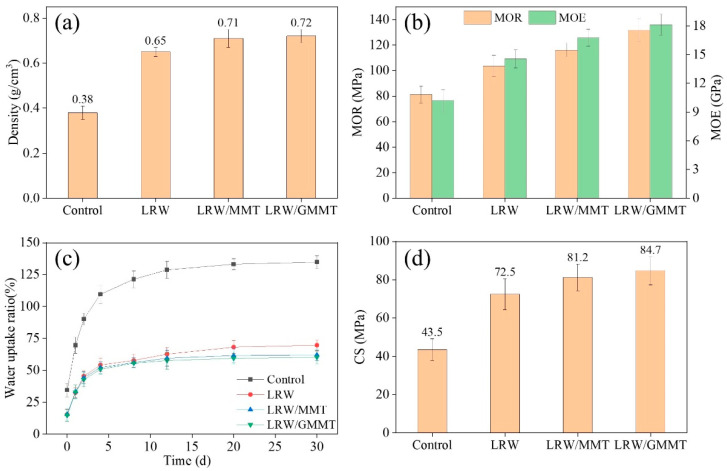
(**a**) Density, (**b**) MOE and MOR, (**c**) WU, and (**d**) CS of the control, LRW, LRW/MMT, and LRW/GMMT.

The impregnation of LR and GMMT into the wood show significant improvements in the wood’s physical and mechanical properties, which can be seen from Figure 3. The density of the control was 0.38 g/cm^3^. After treatment with LR/GMMT, the density of the poplar wood sample increased to 0.72 g/cm^3^. The value of density is crucial to the properties of wood, which could influence physical performance significantly with density increasing. MOR and MOE of the control were 81.2 MPa and 10.21 GPa, respectively. Compared with LRW and LRW/MMT, the LRW/GMMT obtained the largest increase in MOR and MOE after treatment, up to 131.8 MPa and 18.14 GPa, respectively, which was 62.3% and 77.7% higher than the control. The pristine poplar wood has low density and inferior mechanical properties. After treatment, the wood cavities are filled, and the cell structures can be strengthened, resulting in an increase in density and enhancement of mechanical properties [29]. Moreover, the initial WU of the control increased rapidly within 5 days and reached over 100% on the fifth day. At the end of the test, the WU of the control reached 134.8%. After treatment, the WU of treated samples was all reduced, especially the LRW/GMMT. The WU corresponding to the end of the test of LRW/GMMT was 60.3%, illustrating the significant improvement in the water repellency of treated samples. CS of the treated samples was also enhanced significantly. Similar to the trend of MOE and MOR, CS of LRW/GMMT was the most effectively improved. It reached 84.7 MPa, which was 94.7% higher than the control. This may be due to the surface modification of MMT, which has strong hydrophobic properties and stronger compatibility with LR solution after modification [30]. It can be more evenly distributed inside the wood, filling the wood pores and isolating the contact and adsorption with water, thereby significantly reducing the water absorption of poplar wood and improving its mechanical properties.

### 3.3. Analysis of the Reaction Mechanism

The fabrication method of lignin resin is derived from the process of phenolic resin, which applies degraded lignin as an alternative to synthesize the resin. Therefore, the curing process and mechanism of LR are similar to phenolic resin. The curing mechanism could be more complicated with the combination of LR and surface-modified MMT impregnated into the wood structure [31,32].

**Figure 4:**
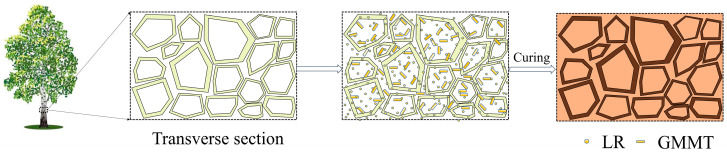
Schematic illustration of hybrid impregnation and curing process for fast-growing poplar wood treatment.

The treating process of poplar wood with hybrid modification is illustrated in Figure 4. In the process of treatment, the hybrid agents, including LR and GMMT, were impregnated into the wood interior, which could permeate and saturate the cell wall structures. Moreover, hybrid agents could also deposit in the cell corner and other wood cavities. Therefore, the wood structures were enhanced by treatment, resulting in the improvement in the comprehensive properties.

**Figure 5:**
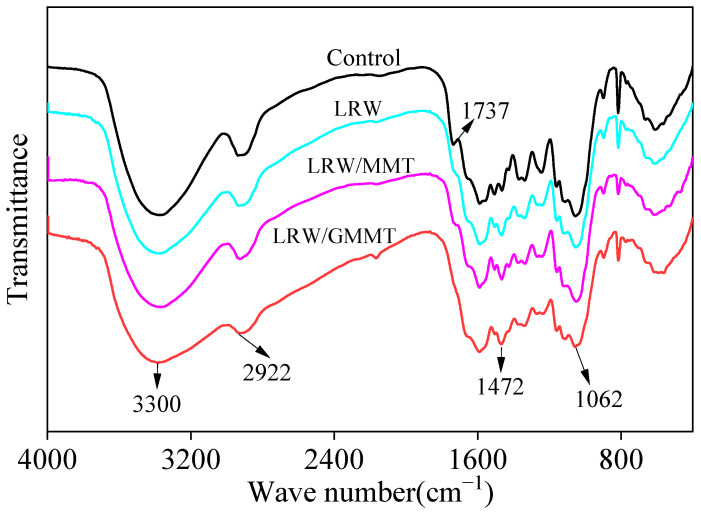
FTIR spectra of different poplar wood samples.

The FTIR spectra of the control, LRW, LRW/MMT, and LRW/GMMT are presented in Figure 5. The characteristic peak of hydroxyl group (–OH) observed at 3300 cm^−1^ could be assigned to the stretching vibration of O–H. After treatment, the peak was weakened, which could be due to the reaction with the LR during the curing process. In addition, the cured LR could cover the surface of the wood cell walls and fill the cell cavities. The hydrophilic groups could be covered by a cured resin layer, resulting in the attenuation of the characteristic peak of –OH. The absorption peak at around 2922 cm^−1^ belonged to the symmetric and asymmetric stretching vibrations of –CH_2_. Similarly, the peak was also weakened after hybrid treatment. This could be attributed to the coverage of the cell walls and cell cavities. The peak observed at 1737 cm^−1^ could be assigned to the carboxyl group. Notably, this peak totally disappeared when the LR was used to treat the wood sample. C–H deformation vibration peak of methylene groups was the characteristic absorption band of the cured PF resin. This peak was also observed in the FTIR spectra of LRW, LRW/MMT, and LRW/GMMT. The bands of 1508 cm^−1^ and 1463 cm^−1^ were finally merged into the peak of 1472 cm^−1^ and strengthened after treatment, indicating the amount of methylene groups was increasing significantly with the curing of LR. Moreover, the C–O stretching vibration of methylol groups was observed at 1062 cm^−1^. With the decrease in hydroxyl groups, the methylol groups were synchronously reduced, leading to the peak weakened after treatment. The results were consistent with the report of Liu et al. [33].

**Figure 6:**
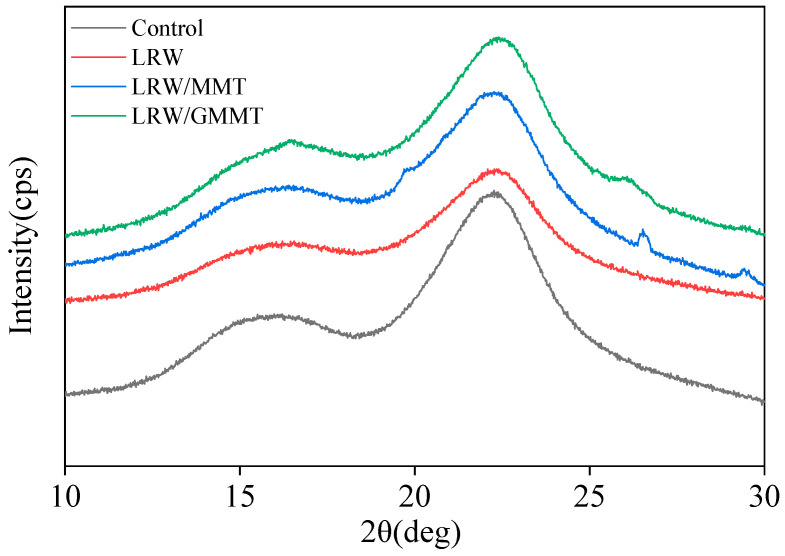
XRD spectra of different poplar wood samples.

The XRD spectra of the control and treated samples are illustrated in Figure 6. The polymerization of LR with GMMT showed a significant effect on the crystallization of treated samples. The diffraction angles at 15.6° and 22.3° of the samples corresponded to the cellulose crystal planes I101 and I002, respectively. The position of the XRD diffraction peaks of the poplar wood samples before and after treatment remained the same, indicating that the treatment of LR and GMMT did not change the crystallization form of poplar wood. However, the XRD diffraction peak intensity of treated wood samples significantly decreased. In addition, the diffraction peaks observed at 26.4° and 29.5° could be assigned to the additions of MMT. However, these two peaks were weakened obviously with the loading of GMMT. The reason could be that the crystallization form of MMT was changed after surface modification. Upon exfoliation, the particle size of GMMT was much smaller than that of pristine MMT. Moreover, some of the chemical groups grafted onto GMMT could react with LR during the heat-curing process. This process could influence and change the crystallization form of the treated poplar wood samples with LR and GMMT as the treating agents.

**Figure 7:**
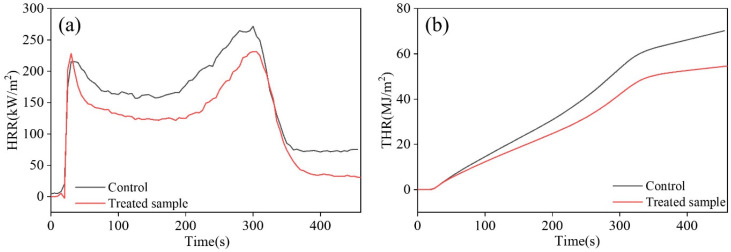
(**a**) The heat release rate (HRR) and (**b**) the total heat release rate (THR) of pristine samples and treated samples prepared under optimal conditions.

The heat release rate (HRR) and total heat release rate (THR) can evaluate the heat release during the material combustion process. By comparing the HRR curves in Figure 7a, it can be seen that around 300 s, the HRR of the control reached its maximum peak while the corresponding peak of LRW/GMMT moved slightly back to 305 s. In addition, the peak HRR of the treated sample was obviously reduced to 231.33 kW/m^2^, a decrease of 17.5% compared to the control (271.71 kW/m^2^). In Figure 7b, it can be seen that the THR of the treated sample (54.77 MJ/m^2^) also significantly decreased compared with that of the control (70.21 MJ/m^2^), indicating that the addition of MMT could effectively reduce the heat release during the burning process.

**Figure 8:**
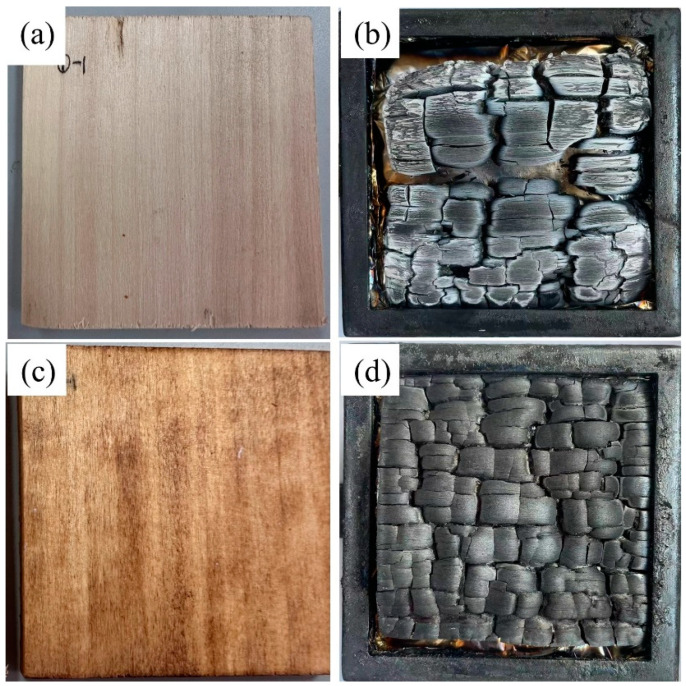
Images of the control (**a**,**b**) and LRW/GMMT (**c**,**d**) before and after CONE test.

The residue and morphology after combustion can be crucial to evaluate the property of flame retardancy. It can be seen that the main structure of the control is almost destroyed by combustion. In addition, there is a large amount of ash on the surface that was burned, with the overall structure incomplete (Figure 8a,b). After treatment, in Figure 8c,d, the overall structure of LRW/GMMT is intact. Moreover, the surface of LRW/GMMT is completely carbonized, with no obvious ash content observed, indicating that the carbonization of treated poplar wood samples is the main process in the combustion process. This is because MMT could degrade into magnesium oxide, aluminum oxide, and silicon oxide, which could form an inorganic layer that blocks combustion at the combustion interface. The degradation process of MMT could especially release a large amount of water, effectively reducing the temperature of the combustion interface.

## 4. Conclusions

In this work, the lignin-derived PF and surface-modified MMT were successfully applied to poplar wood treatment. The treating agents could penetrate the wood, fill the cavities of the wood interior, and strengthen the cell wall structure. The optimal WPG of 36.2% was obtained upon the RSM experiments at the conditions of 34% LR, 1.8% GMMT, 1.2 MPa impregnation pressure, and 99 min impregnation time. The density, WU, MOR, MOE, and CS of the samples were tested to evaluate the enhancement of the physical and mechanical properties. In addition, these samples were investigated via CONE, XRD, and FTIR. The results showed that the density of treated samples increased significantly up to 0.72 g/cm^3^. Compared with 134.8% of the control, the WU of the treated wood sample could decrease to 60.3%. In addition, the MOR and MOE of the resulting samples reached up to 131.8 MPa and 18.14 GPa, respectively, which were 62.3% and 77.7% higher than the control. Notably, the CS was 84.7 MPa, with an increase of up to 94.7%. In addition, the peak HRR of the treated sample was obviously reduced to 231.33 kW/m^2^, a decrease of 17.5% compared to the control (271.71 kW/m^2^). The overall structure of the treated sample is intact, with the surface completely carbonized.

## Figures and Tables

**Table 1:** The response surface of factor level and coding.

Variables	Levels		
LR (A, wt%)	25	30	35
GMMT (B, wt%)	1.5	2	2.5
Impregnation pressure (C, MPa)	0.9	1.1	1.3
Impregnation time (D, min)	80	90	100

**Table 2:** The experiment results of BBD.

Number	LR (A, wt%)	GMMT (B, wt%)	Impregnation Pressure (C, MPa)	Impregnation Time (D, min)	WPG (Y, %)
1	35	2	1.3	90	29.5
2	30	2	1.1	90	35.8
3	30	1.5	1.1	100	31.8
4	30	2	1.3	100	35.7
5	25	2	1.1	80	31.7
6	30	2.5	1.1	100	33.2
7	30	2	0.9	100	34.1
8	35	2	1.1	100	35.4
9	30	2	1.1	90	29.1
10	30	2	1.1	90	35.5
11	30	1.5	0.9	90	31.2
12	30	2	1.3	80	36.1
13	30	2.5	1.1	80	31.5
14	35	1.5	1.1	90	32.8
15	25	2	0.9	90	33.0
16	30	2	1.1	90	35.7
17	25	2	1.1	100	26.3
18	35	2	1.1	80	35.1
19	30	1.5	1.3	90	31.7
20	35	2.5	1.1	90	36.0
21	30	2.5	1.3	90	31.6
22	30	2	1.1	90	33.5
23	30	1.5	1.1	80	34.3
24	25	2.5	1.1	90	35.8
25	35	2	0.9	90	33.6
26	30	2	0.9	80	33.7
27	30	2.5	0.9	90	33.6
28	25	2	1.3	90	33.7
29	25	1.5	1.1	90	34.5

**Table 3:** The ANOVA of response surface.

Source	Sum of Square	Df	Mean Square	F-Value	*p*-Value	Significance *
Model	149.64	14	10.69	30.20	<0.0001	**
A	99.76	1	99.76	281.85	<0.0001	**
B	7.68	1	7.68	21.70	0.0004	**
C	15.19	1	15.19	42.91	<0.0001	**
D	12.61	1	12.61	35.62	<0.0001	**
AB	1.44	1	1.44	4.07	0.0633	
AC	5.06	1	5.06	14.30	0.0020	**
AD	0.56	1	0.56	1.59	0.2281	
BC	0.49	1	0.49	1.38	0.2590	
BD	0.040	1	0.040	0.11	0.7417	
CD	1.000 × 10^−2^	1	1.000 × 10^−2^	0.028	0.8689	
A^2^	4.89	1	4.89	13.82	0.0023	**
B^2^	6.505 × 10^−3^	1	6.505 × 10^−3^	0.018	0.8941	
C^2^	1.83	1	1.83	5.16	0.0394	*
D^2^	0.058	1	0.058	0.16	0.6930	
Residue	4.96	14	0.35			
Lack of fit	4.37	10	0.44	2.97	0.1527	
R^2^	0.9679					

* Significant at the 5% level (*p* < 0.05); ** Significant at the 1% level (*p* < 0.01).

## Data Availability

Not applicable.
